# Robotic Ultrasound Scanning End-Effector with Adjustable Constant Contact Force

**DOI:** 10.34133/cbsystems.0251

**Published:** 2025-05-02

**Authors:** Zehao Wu, Xianli Wang, Yuning Cao, Weijian Zhang, Qingsong Xu

**Affiliations:** Department of Electromechanical Engineering, Faculty of Science and Technology, University of Macau, Taipa, Macau, China.

## Abstract

In modern medical treatment, ultrasound scanning provides a radiation-free medical imaging method for the diagnosis of soft tissues via skin contact. However, the exerted contact force heavily relies on the skill and experience of the operator, which poses great inspection instability. This article reports on a robotic ultrasound scanning system with a constant-force end-effector. Its uniqueness is the introduction of a hybrid active–passive force control approach to maintaining a constant contact force between the ultrasound probe and the continually changing surface. In particular, the passive constant-force mechanism provides strong buffering to the force variation. The active force control system improves flexibility and provides long-stroke positioning. Experimental tests on both silicone models and human volunteers demonstrate the capability of the proposed robotic ultrasound scanning system for obtaining qualified ultrasound images with high repeatability. Moreover, the ease of operation of the robotic US scanning system is verified. This work provides a promising method to assist doctors in conducting better and cushier ultrasound scanning imaging.

## Introduction

Medical imaging technology is an important technique in modern medical treatment. It provides an essential diagnostic basis for clinical therapy from early diagnosis to the final schedule of the best treatment procedures [1–3]. To date, standard medical imaging technologies in clinical treatment include digital radiography, computed tomography, magnetic resonance imaging, and ultrasound (US) imaging. In particular, US imaging has merits in terms of being harmless, low cost, real-time imaging, high portability, and no radiation, which make it the first choice of frequent imaging for soft tissues [4,5]. Hence, it has been widely used in the clinical diagnosis of heart [6], obstetrics and gynecology [7], abdomen [8,9], small organs [10], and blood vessels [11]. Recent advancements in US technology have led to the development of innovative wearable bioadhesive US devices [12,13], which enable continuous, real-time monitoring of pathological lesions. These compact, conformable systems demonstrate great potential for longitudinal assessment of disease progression and treatment efficacy. Meanwhile, conventional US probes remain indispensable in clinical practice, which are primarily employed by medical professionals for comprehensive lesion screening and large-area diagnostic examinations. In addition, the US probes have wide applications in in vivo imaging of untethered medical robots [14–16]. In traditional manual US imaging with US probe, obtaining a high-quality US image is challenging, requiring the operator to have rich experience, superb techniques, and sensitive visual and tactile senses [17]. Therefore, manual US imaging can easily cause misdiagnosis, which is challenging to apply to clinical treatment, demanding repeated biological measurements and reproducible images to monitor lesion conditions [18,19].

**Fig. 1.**
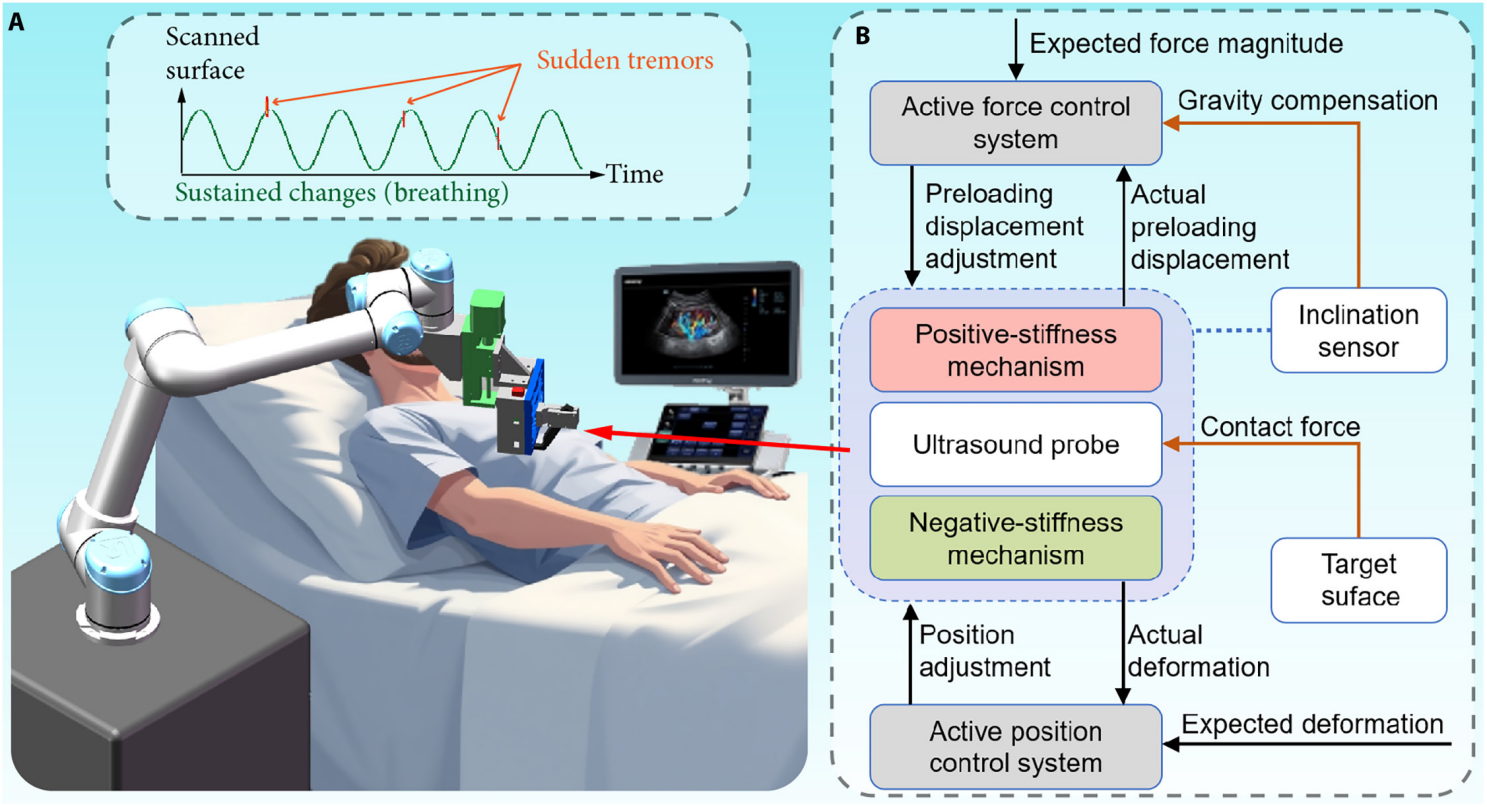
Schematic of the robotic ultrasound (US) scanning system. (A) Robotic arm for positioning the hybrid active–passive force control end-effector (HFCE) to the target scanned surface of the patient. (B) Flowchart of the working principle of the HFCE.

To overcome the limitations of manual US imaging and to reduce the heavy operational tasks for the operator, robots can be utilized to assist the US imaging due to their high stability and repeatability (Fig. 1A) [20,21]. The contact force between the US probe and the scanned surface mainly influences the quality of the obtained US image [22]. An insufficient contact force will cause poor acoustic coupling, which makes it impossible to display the scanning tissue clearly. On the contrary, an excessive contact force will change the shape of the scanning tissue, which may cause misdiagnosis. Then, a continuous variation of contact force will lead to a continuous change in tissue deformation, which in turn results in observable oscillations in the US image pattern. Therefore, maintaining a suitable contact force during the US scanning can effectively improve the quality and repeatability of the US image. However, it is difficult for a human operator to achieve this [17]. Thus, it is an essential research focus for the robotic US scanning system [23,24].

In the conventional contact-force control system, we can regulate the magnitude of contact force by resorting to the feedback of force sensing or an accurate force estimation model called active force control. For example, Merouche et al. [25] utilized a 6-axis force/torque sensor to maintain a constant contact force on the scanned surface. Gilbertson and Anthony [26] designed a handheld force-controlled US probe based on a hybrid force-position control, which can reduce the force variation by 83% with 1 mm and 1 Hz of hand oscillation. Ma et al. [27] predicted the required adjustment of the pressing depth based on the information from the depth camera and force sensor. However, since the environment is unknown and unpredictable in practical application, the overshoot of the conventional active force control may threaten patient safety. Thus, researchers have proposed to use active compliant force control methods to regulate the contact force, e.g., admittance control [28] and impedance control [29]. Through the control algorithm, the compliant force control methods can achieve soft landing of the US probe on the scanned surface. However, for the US scanning, obtaining an accurate control model is difficult due to the personal differences among the patients. This limits the practical application of compliant force control methods on the US scanning. In addition, the scanned objectives are not static, and the scanned surface continuously changes due to the patient’s physiological activity (such as breathing). The interference from the sudden tremor of the patient is also worth noting. Therefore, to ensure the stability of robotic US scanning and the safety of patients, researchers usually adopt an expensive force sensor to provide a high rate of force feedback and a positioning actuator to provide high control bandwidth and positioning accuracy [30,31]. Meanwhile, an additional spring between the actuator and the US scanning probe can further improve the performance of the force control system because it buffers the amplitude of contact force variation with displacement [32,33].

An alternative approach to maintaining a suitable contact force is passive force control. The specially designed springs or compliant mechanisms provide a specified stroke region with constant force (i.e., zero stiffness). Then, a quick generation of the constant force can be accomplished by deforming the mechanism within its stroke region under an open-loop motion control. In addition, the control bandwidth of the passive force control can be defined as infinitely large. For example, Tsumura and Iwata [34] proposed a passive US scanning system for observing fetal body parts integrated with a constant-force spring. Housden et al. [35] designed a spring-ball-based mechanical clutch to limit the maximum contact force. Moreover, some compliant mechanism-based constant-force springs were proposed to control the contact force in the literature [36–38]. The compliant mechanism can effectively reduce the force variation owing to its merits of no wear and no gap. The advantages of passive force control include no overshoot, simple control, and low cost. However, it has the drawback of high specificity, which is unsuitable for a batch of tasks with many uncertain factors. In addition, the suitable contact force is not a fixed value due to the personal difference. Therefore, the magnitude of the contact force should be adjustable among the patients.

To overcome the limitations of the existing force control approaches, this article achieves constant-force US scanning by proposing an active control system integrated with a passive constant-force mechanism, i.e., hybrid active–passive force control end-effector (HFCE) (Fig. 1B). The whole robotic US scanning system is composed of the proposed HFCE, haptic interface, and robotic arm, where the teleoperation system can ease the burden of human operators. The passive force control is obtained by parallel collection of a positive-stiffness mechanism and a negative-stiffness mechanism. The constant-force region of the passive constant-force mechanism can buffer a sudden trembling to effectively compensate for the limited control bandwidth of the active control system, which reduces the requirement for a force sensor and positioning actuator. Moreover, the active control system is adopted to adjust the magnitude of the constant force of the passive mechanism. It also compensates for the gravity effect and adjusts the deformation of the passive mechanism, which overcomes the deficiency of high specificity for the passive force control. Experimental results demonstrate that the proposed HFCE can tolerate more considerable interference from sustained changes and sudden tremors. The reported US scanning system with the HFCE can assist doctors in obtaining a qualified and repeatable US image to focus more on diagnosis by simplifying their heavy operational tasks of US scanning.

## Materials and Methods

### Mechanism design and simulation studies

**Fig. 2.**
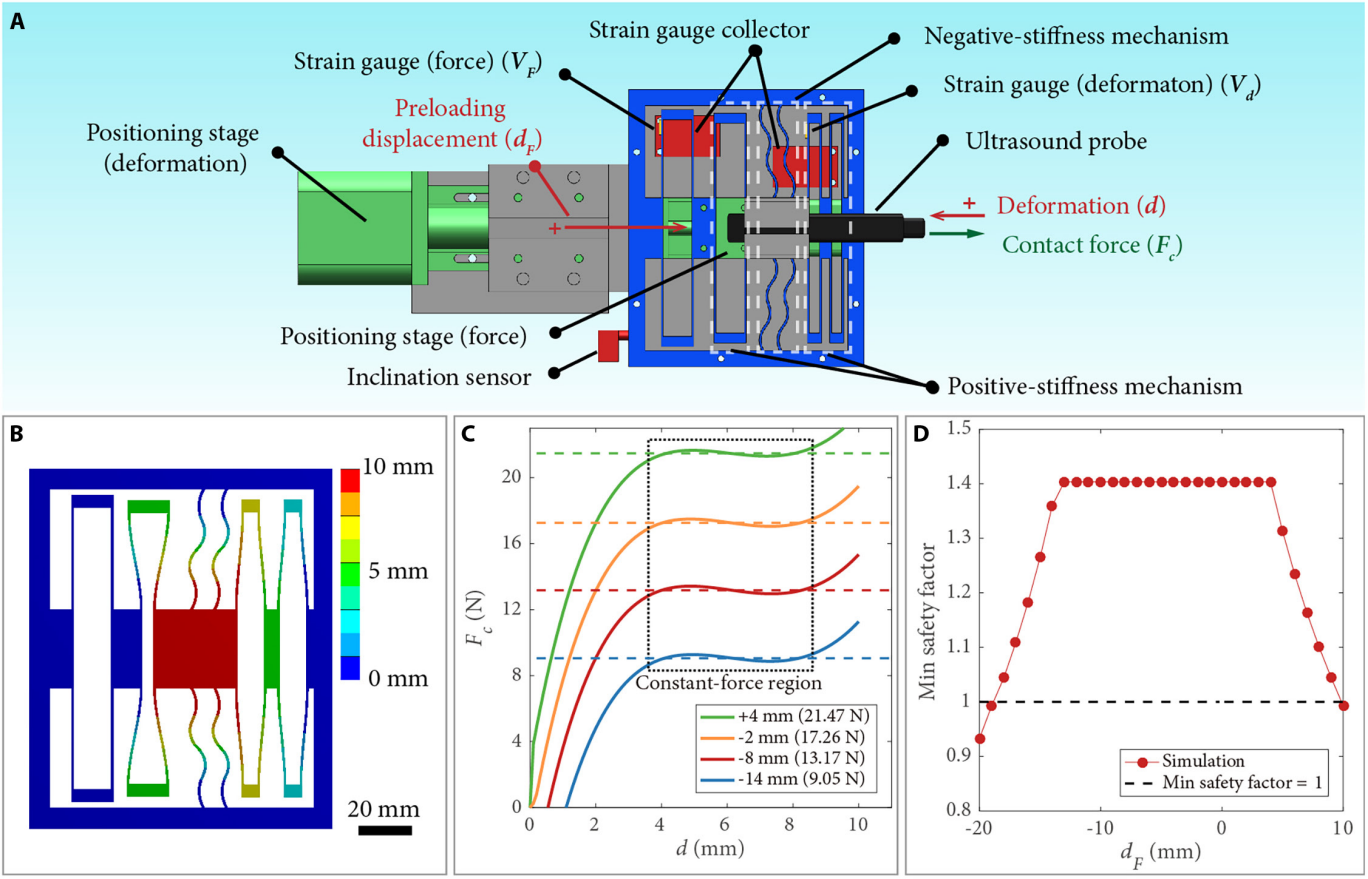
Mechanism design and simulation results of HFCE. (A) Schematic of HFCE. (B) Deformation distribution of the passive constant-force mechanism without a preloading displacement. (C) Force–deformation curves of the passive constant-force mechanism with various preloading displacements. (D) Minimal safety factors of the passive constant-force mechanism versus the preloading displacement ($d_{F}$).

The mechanism design of the proposed HFCE is shown in Fig. 2A. $F_{c}$ and d denote the contact force and deformation of the passive constant-force mechanism, respectively. The proposed HFCE consists of 2 parts: the passive constant-force mechanism and the active force control system. The passive constant-force mechanism is realized by combining a positive-stiffness and a negative-stiffness mechanism. The positive-stiffness mechanism comprises several straight beams connected in series or parallel, providing a linear positive stiffness. Four parallel-connected tilted curve beams construct the negative-stiffness mechanism. In addition, the curved beam structures have superior deformation capacity compared to conventional straight beams. This enhanced deformation capability significantly expands the constant-force region. According to the buckling effect, a part of the deformation exhibits negative stiffness, which mainly determines the length of the constant-force region of the passive constant-force mechanism. By superimposing the magnitudes of positive stiffness and negative stiffness, a constant-force region with zero stiffness is generated to provide the constant contact force ($F_{\text{const}}$) (Fig. S1). Moreover, considering the convenience, cost, and manufacturing capability for complex structures, 3-dimensional (3D) printing is chosen as the production method of passive constant-force structure, and the common acrylonitrile butadiene styrene (ABS) elastic material is used as the mechanism material.

Furthermore, simulation studies were conducted using static structural analysis using ANSYS software. The outer edge is designated as the fixed support. Displacement supports were applied to create the preloading displacement for the deformation of the positive-stiffness mechanism and main moving part, respectively. The reaction force of the moving part was measured as the contact force. Parameter optimization was carried out to identify the appropriate structural parameters. The rough constant-force region of the constant-force mechanism was determined according to the simulation result of the passive constant-force mechanism with rough structural parameters. Consequently, the maximum and minimum contact forces within this rough constant region were measured. Then, the force variation is derived within this region. The genetic algorithm was chosen for optimization. The primary optimization objective was to achieve a constant force in the designated motion region with minimized force variation. In addition, the minimal safety factor during this region should be larger than 1.0 to avoid material failure. The optimization results are shown in Table S1.

The deformation distribution of the passive constant-force mechanism is shown in Fig. 2B. In practice, the US scanning probe is connected to the main moving part of the passive constant-force mechanism (red part in Fig. 2B). Moreover, one terminal of the positive-stiffness mechanism is used to adjust the magnitude of the constant force and to compensate for the gravity effect. By applying a preloading displacement ($d_{F}$) on this terminal (Fig. 2A), the force–deformation curve of the positive-stiffness mechanism can shift upward or downward, which changes its combined magnitude of the constant force (Fig. S1). Fig. 2C gives the simulation results of force–deformation curves of the passive constant-force mechanism under various preloading displacements, which confirm the effectiveness of adjustable constant contact force and active gravity compensation. In addition, to avoid material failure, the minimal safety factor under various preloading displacements is obtained by simulation study, and the results are displayed in Fig. 2D. To keep the safety factor larger than 1.0, the maximum tuning range of the preloading displacement varies from –18 to 9 mm. In this study, we select the tuning range of preloading displacement from –14 to 4 mm, in which the safety factor of 1.4 is not obviously reduced.

The active force control system is developed by using an inclination sensor, 2 strain gauges, and 2 positioning stages. The inclination sensor is adopted to measure the inclination angle (θ) between the HFCE and the gravitational direction, which is employed to determine the magnitude of gravity compensation. The 2 strain gauges are adopted to measure the real-time deformation and preloading displacement of the proposed constant-force mechanism (Fig. 2A). The measured voltage increase and decrease relative to the initial values of these 2 strain gauges are denoted as $V_{d}\;$ and $V_{F},$ respectively. In addition, for real-time measurement of the preloading displacement, additional straight beams are linked between the force-adjusting terminal and an outside framework. Each positioning stage comprises a step motor, a linear slide rail, and a slider. Based on the feedback provided by the 2 integrated strain gauges and the inclination sensor, the required driving step numbers of both step motors can be determined. Then, the positions are obtained at their fastest stepping speeds.

### Fabrication

The passive constant-force mechanism was fabricated by 3D printing using ABS material. The positioning stage for adjusting the magnitude of the constant force and the deformation of the passive constant-force mechanism are CTM28 and CB42 from Beijing Haijie Jiachuang Technology Co. Ltd., offering positioning ranges of 20 and 50 mm, respectively. The strain gauges (model: BF350-3AA, from Shenzhen Luojia Technology Co. Ltd., China) provide a resolution of 0.02 V (i.e., 0.35 mm for displacement d and 0.40 mm for displacement $d_{F}$) in the control system. The model of the inclined sensor is MPU6050. The US system (Terason u-smart 3300, from Teratech) was used to collect the US images. The control board utilized in the active control system is Arduino Mega2560. A robot arm (UR5, from Universal Robots A/S) was adopted to move the HFCE to the targeting surface, controlled by a haptic interface (model: Geomagic Touch, from 3D Systems Corp.).

### Experimental equipment

The passive constant-force mechanism was positioned by a 3D position stage (LPAA301, from Beijing Lepu Technology Co. Ltd.). A commercial load cell (LSB200-25lb, from FUTEK Advanced Sensor Technology Inc.) was adopted to measure the contact force. The displacement was measured by a laser displacement sensor (LK-H055, from Keyence Corp.). A shaker (KSI-758ST150, from KingSci Instruments Inc.) generated the vibration. The material of the custom-built silicone sample was Dragon Skin 10 (from About Smooth-On Inc.). The data were collected by a real-time controller (NI PXIe-8881, from National Instruments Inc.).

### Teleoperation system of the robotic arm

The teleoperation system comprises 2 parts: position mapping and orientation mapping. Each part is activated by pressing the corresponding button on the haptic interface. Once activated, the position or orientation change will control the robotic arm to give the corresponding change with the predefined gain.

### Demonstration of robotic US scanning system

Approval of all ethical and experimental procedures and protocols was granted by the Research Ethics Committee of the University of Macau under application no. APP-ARE-057. Volunteers signed informed consent forms before participating in this study.

## Results

### Characterization of the passive constant-force mechanism

**Fig. 3.**
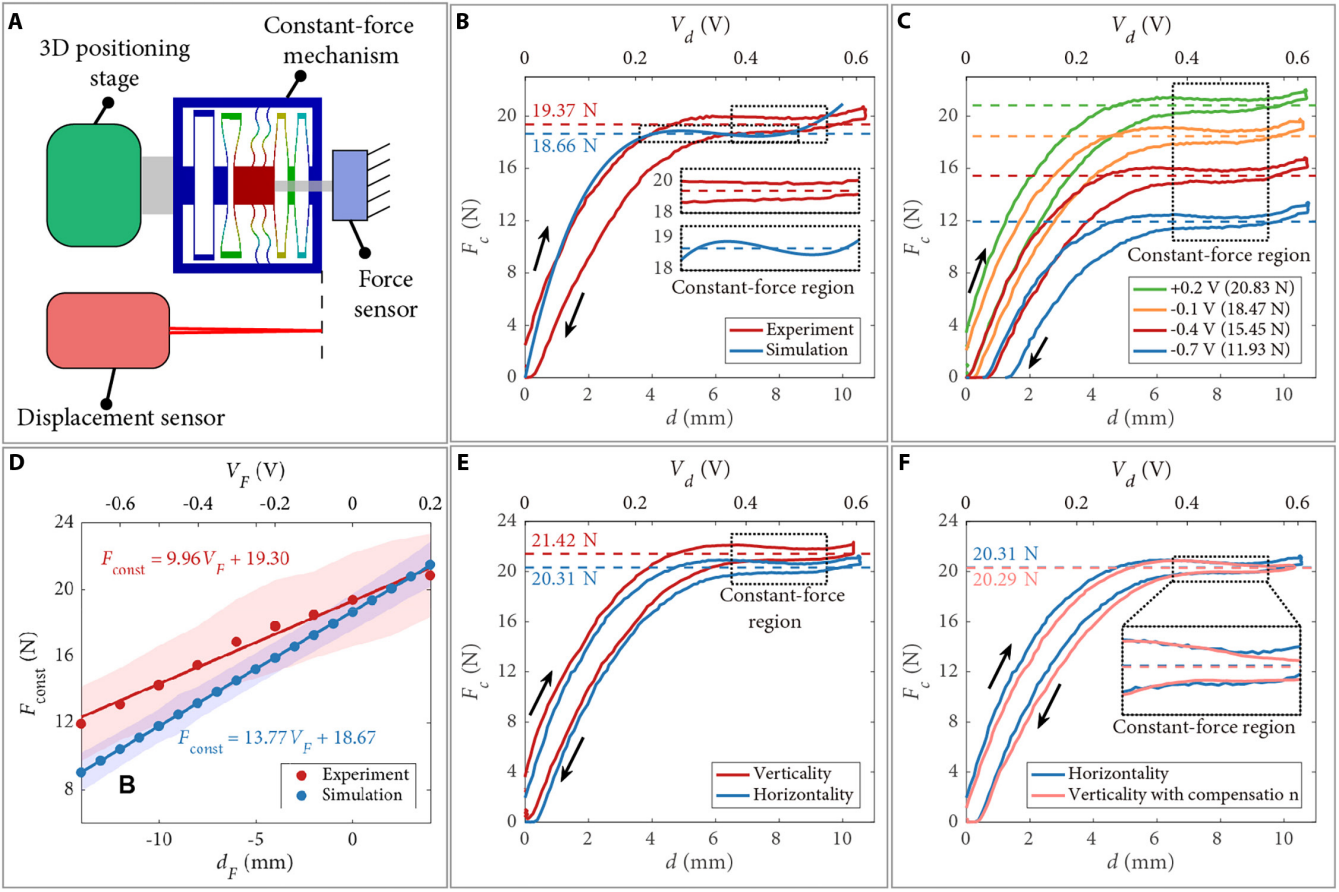
Experimental results of the passive constant-force mechanism. (A) Experimental setup. (B) Experimental and simulation results of the force–deformation curves without preloading displacement. (C) Force–deformation curves at various preloading displacements. (D) Experimental and simulation results of the relationships between the magnitude ($F_{\text{const}}$) of constant force and the preloading displacement ($d_{F}$). Solid line: fitting line. Shaded area: force variation in the constant-force region. Force–deformation curves with a preloading displacement of +2 mm under the vertical and horizontal direction (E) without or (F) with gravity compensation. Black arrow: deforming direction. Dashed line: mean value in constant-force region.

To demonstrate the capabilities of the passive constant-force mechanism, we obtained the force–deformation relationship of the passive constant-force mechanism at various conditions, as shown in Fig. 3A. In addition, the relationships between the measured voltage outputs of strain gauges and the corresponding deformations are given in Fig. S2. The results illustrate the signal voltages of the strain gauges at the position $\left(V_{d}\right)$ with various d and the constant force $\left(V_{F}\right)$ with different $d_{F}$.

To examine the difference between the simulation result and actual capability, a comparison study is carried out between the simulation and experimental results for the passive constant-force mechanism without preloading displacement, as shown in Fig. 3B. It is found that due to the manufacturing error and assembly error, the starting position of the constant-force region shifts backward (from 3.6 to 6.5 mm), and its length is decreased (from 5 to 3 mm). Moreover, there is a hysteresis loop between the compressive deformation and deformation recovery in the experimental result, which is caused by the material adopted for fabrication. The hysteresis mainly causes the increase of the force variation in the constant-force region. Compared to the simulation result without preloading displacement, the force variation is increased from 3.40% to 7.20%. Meanwhile, the magnitude error of the constant force is derived as 3.67%, which is acceptable.

Next, to characterize the relationship between the magnitude of constant force and preloading displacement, the force–deformation curves at various preloading displacements were measured under horizontal conditions, as shown in Fig. 3C and Fig. S3. The magnitudes of constant force and corresponding curve fitting are given in Fig. 3D. The results indicate that adjusting the magnitude of constant force is effective by tuning the preloading displacement. The adjustment range and ratio of the constant force are derived as [12.33 N, 21.29 N] and (9.96 N/V)$V_{F}$, respectively. In addition, the actual adjustment range is 0.72 times the simulation result. Such results provide a reference for the initial setting of the active force control system. The expected deformation is assigned as the middle position of the constant-force region (i.e., 8 mm, $V_{d}=+$0.46 V). Then, the actual magnitude of the constant force can be derived from $V_{F}$ value.

To further eliminate the gravity effect and to achieve stable multiple-angle US scanning, the influence of gravity on the magnitude of constant force is measured, as shown in Fig. 3E. It is observed that without compensation, the magnitude of constant force under vertical (in gravitational) direction is 1.11 N larger than that in the horizontal direction. Then, the compensation for the gravity effect is determined as (–1.11 N)cosθ. After gravity compensation, the magnitudes of constant force under vertical and horizontal directions are consistent (Fig. 3F).

### Characterization of the vibration isolation capability of HFCE

**Fig. 4.**
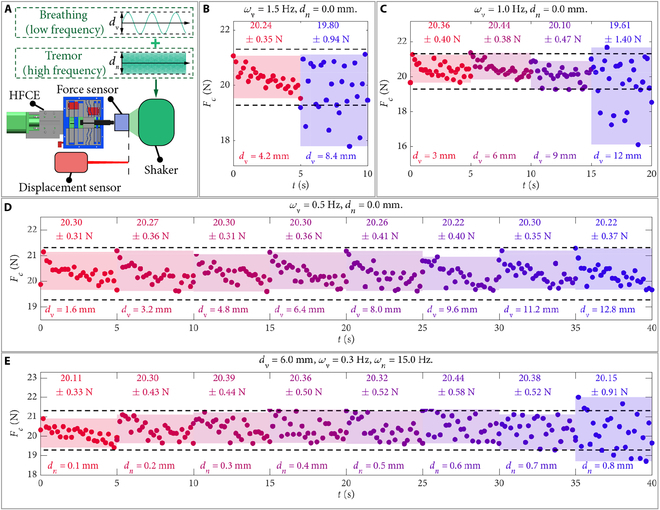
Vibration isolation capability test results of HFCE. (A) Experimental setup. Experimental results of the contact force under (B) 1.5 Hz, (C) 1.0 Hz, and (D) 0.5 Hz vibrations at various vibration magnitudes ($d_{v}$) without an input noise. (E) Experimental results of the contact force under the vibrations with various noise magnitudes ($d_{n}$), vibration of 6 mm and 0.3 Hz, and noise frequency of 15 Hz. Black circle: experimental data. Dashed line: desired contact force range. Shaded area: experimental data range. Top text: mean value ± standard deviation (SD).

After demonstrating the performance of the passive constant-force mechanism, we assembled the whole HFCE to investigate its capability to maintain a constant contact force under various vibration conditions (Fig. 4A). The shaking of vibration is described as $d_{v}\text{sin}\left(2\pi f_{v}t\right)/2+d_{n}\text{sin}\left(2\pi f_{n}t\right)/2$, where t is the time. The peak–peak magnitudes of the vibration and noise (sudden tremors) are determined as $d_{v}$ and $d_{n}$, respectively. The frequencies of the vibration and noise (sudden tremors) are derived as $f_{v}$ and $f_{n},$ respectively.

First, without an input noise, we investigated the influence of vibration frequency and magnitude on the contact force (Fig. 4B to D). At a smaller vibration frequency, the allowed magnitude of vibration increases (Fig. 4B and C). Moreover, the average vibration velocities of the unstable vibration magnitude are derived as 25.2 and 24.0 mm/s, respectively. These 2 velocities are larger than the maximum moving speed (17.2 mm/s) of the positioning stage for adjusting the deformation under setting conditions. This confirms the effectiveness of deformation buffering by the constant-force region of the passive constant-force mechanism. In addition, Fig. 4D shows that the contact force from the HFCE can maintain stability under 0.5 Hz vibration with a peak–peak magnitude up to 12.8 mm, indicating that the proposed HFCE can tolerate the common vibration induced by breathing. For reference, the frequency of human breathing is around 12 to 20 times per minute. Under resting respiratory conditions, the abdominal excursion amplitude is maintained within the range of 2 to 5 mm.

Next, to demonstrate the capabilities of vibration isolation to sudden tremors, the contact force of the HFCE was measured under the shaking with a magnitude of (3 mm)$\text{sin}\left(1.88t\right)+$$d_{n}\text{sin}\left(94.2t\right)$, which represents a vibration frequency ($\omega_{v}$) of 0.3 Hz with a sudden noise frequency ($\omega_{n}$) of 15 Hz. Fig. 4E indicates that when the peak–peak magnitude of the noise is less than 0.7 mm, the contact force is highly stable, illustrating the capability of the HFCE under high frequency and low magnitude of sudden interference. In addition, the average vibration velocity of 0.8 mm noise is derived as 24 mm/s. Therefore, the maximum allowable average vibration velocity is determined as 21 mm/s, corresponding to 0.7 mm noise in Fig. 4E.

**Fig. 5.**
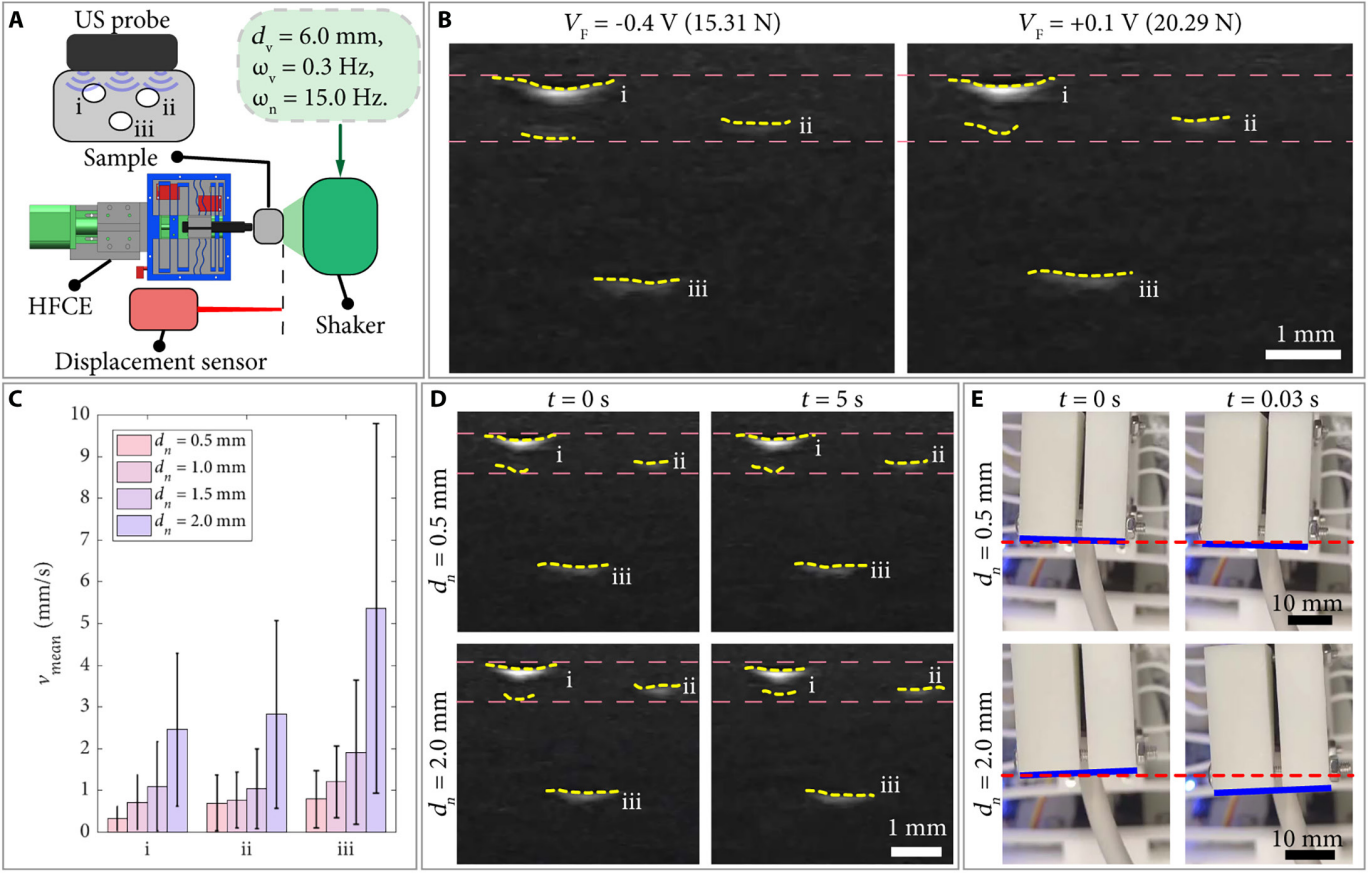
US imaging test results of HFCE. (A) Experimental setup. (B) US images of various contact forces acting on the silicone sample under 0.5-mm noise. (C) Mean velocities ($v_{\text{mean}}$) ± SD of the measured centers of 3 channels under various noise magnitudes. (D) US images at various noise magnitudes. (E) Snapshots of the main moving part at various noise magnitudes. The vibration has 6-mm magnitude and 0.3-Hz frequency, with a noise frequency of 15 Hz. Yellow dashed curve: labeled edge of the channel. Pink/red dashed curve: position contrast line. Blue curve: labeled edge of the main moving part.

For demonstrating the performance of HFCE on US imaging, we took US imaging of 3 channels inside a custom-built silicone model under vibration isolation of sudden tremors (Fig. 5A). The shaking can be expressed as (3 mm)$\text{sin}\;1.88t+d_{n}\text{sin}\;94.2t$. Fig. 5B and Movie S1 show the US images under different contact forces under 0.5 mm noise. It is observed that both magnitudes of contact force kept stable under interference to maintain the quality of collected US images. Moreover, Fig. 5B shows that the compression force of channel i under 20.29 N is larger than that under 15.31 N, and the position of the channel under 20.29 N is higher than that under 15.31 N. These results verified the influence of contact force on the US image.

To further confirm the influence of non-isolated noise on US images, the centers of the 3 channels during the vibrations with different magnitudes of noise are measured by computer image processing. Specifically, each frame of the movie sequence is preprocessed by binarization based on adaptive threshold, and the target regions of 3 channels are segmented accurately. Then, the target region’s centroid coordinates of the binary images are respectively calculated for the channels. Furthermore, the time series analysis is carried out on the centroid coordinates between the continuous frames, and the velocities of the channels are derived by the central difference method. The stability of US images is characterized by the mean velocities of the centers of 3 channels, as shown in Fig. 5C.

For a visual illustration, the selected US images and snapshots of the experiment are shown in Fig. 5D and E, respectively. It is observed that the mean velocities of the centers of 3 channels increase with the magnitude of noise, indicating that the stability of US image reduces as the increasing magnitude of noise. Moreover, Fig. 5C shows that the velocity has a large standard deviation (SD) caused by the periodic intense oscillations and stable images, as shown in Movie S2. Through observation and analysis, the images under intense oscillation are obtained when the main moving part is outside the constant-force region. Moreover, the images remain stable when the main moving part oscillates through the constant-force region. Therefore, the state of images exhibits periodic properties. In addition, the difference of mean velocities between 0.5 and 1.5 mm noises is slowly increasing, which may result from the silicone model’s softness (vibration isolation).

### Construction of the robotic US scanning system

**Fig. 6.**
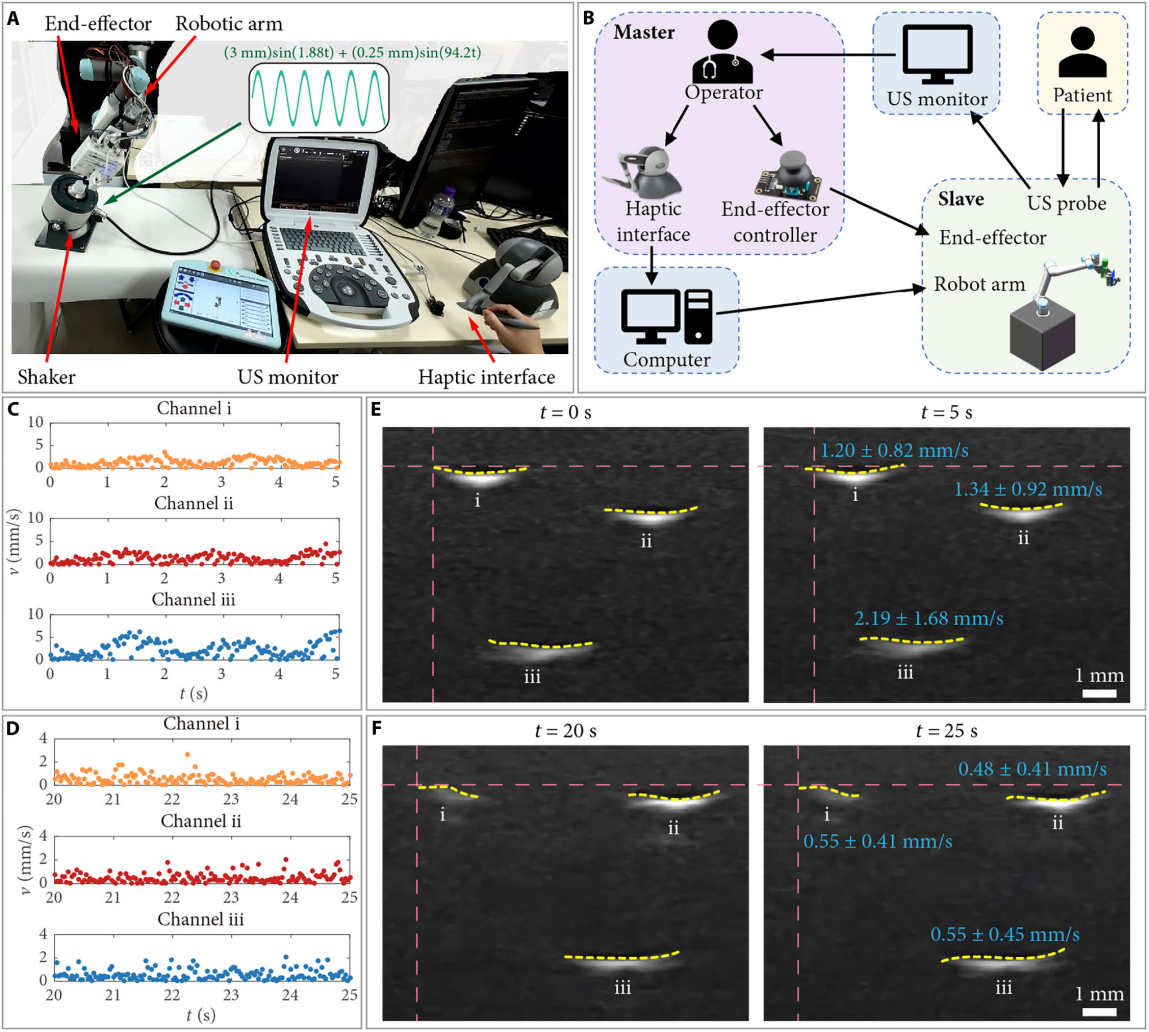
Construction and testing of the robotic US scanning system. (A) Experimental setup. (B) Connection scheme of the robotic US scanning system. Tracking results of the velocity (v) of the measured centers of 3 channels (C) without and (D) with using robotic US scanning system. (D) US images during the scanning (E) without and (F) with using robotic US scanning system. The vibration has 6-mm magnitude and 0.3-Hz frequency, with a noise frequency of 15 Hz. Yellow dashed curve: labeled edge of the channel. Pink dashed curve: position contrast line. Blue text: mean velocity ± SD.

To showcase the utility of the proposed HFCE, it is integrated into a collaborative robotic arm (Fig. 6A). To reduce the learning cost of the human operator. A haptic interface optionally maps the operator’s hand movement onto the robotic arm. Once it reaches the target surface, the operator presses the end-effector controller to turn on the HFCE. Then, the HFCE automatically maintains the contact force at the operator’s expected magnitude. As a result, the US image is kept stable. Fig. 6B shows the structure of the developed robotic US scanning system.

To further confirm the ease operation of the proposed robotic US scanning system, an experience-free operator was invited to use the system to detect the silicone sample on the shaker (Movie S3). The set force magnitude is 20.29 N ($V_{F}=+$0.1 V). The shaking can be expressed as (3 mm)$\text{sin}\left(1.88t\right)$ + (0.25 mm)$\text{sin}\left(94.2t\right)$. Meanwhile, the surface of the silicone sample is 25° tilted relative to the horizontal plane. For comparison, the operator also detected the silicone sample manually. Fig. 6C and D shows the tracking results of the velocities (v) of the measured centers of 3 channels by manual operation and using the proposed robotic US scanning system during a period of 5 s, respectively. The results indicate that the collected US image by the proposed robotic US scanning system is more stable than the manual operation by the experience-free operator. In addition, under shaking, it is found that the collected US images are easy to occur shifting under manual operation (Fig. 6E). Moreover, during the period of 20 to 25 s in Movie S3, the mean velocities of the measured centers of 3 channels using the proposed robotic US scanning system are derived as 0.53, 0.49, and 0.56 mm/s, respectively. These values are close to the mean magnitude of the measured image oscillation under the same shaking in Fig. 6C (0.55 mm/s) and much lower than the magnitudes with manual operation. The results demonstrate that the proposed HFCE can effectively assist the US scanning and reduce the operator’s learning cost.

### Demonstration of the robotic US scanning system

**Fig. 7.**
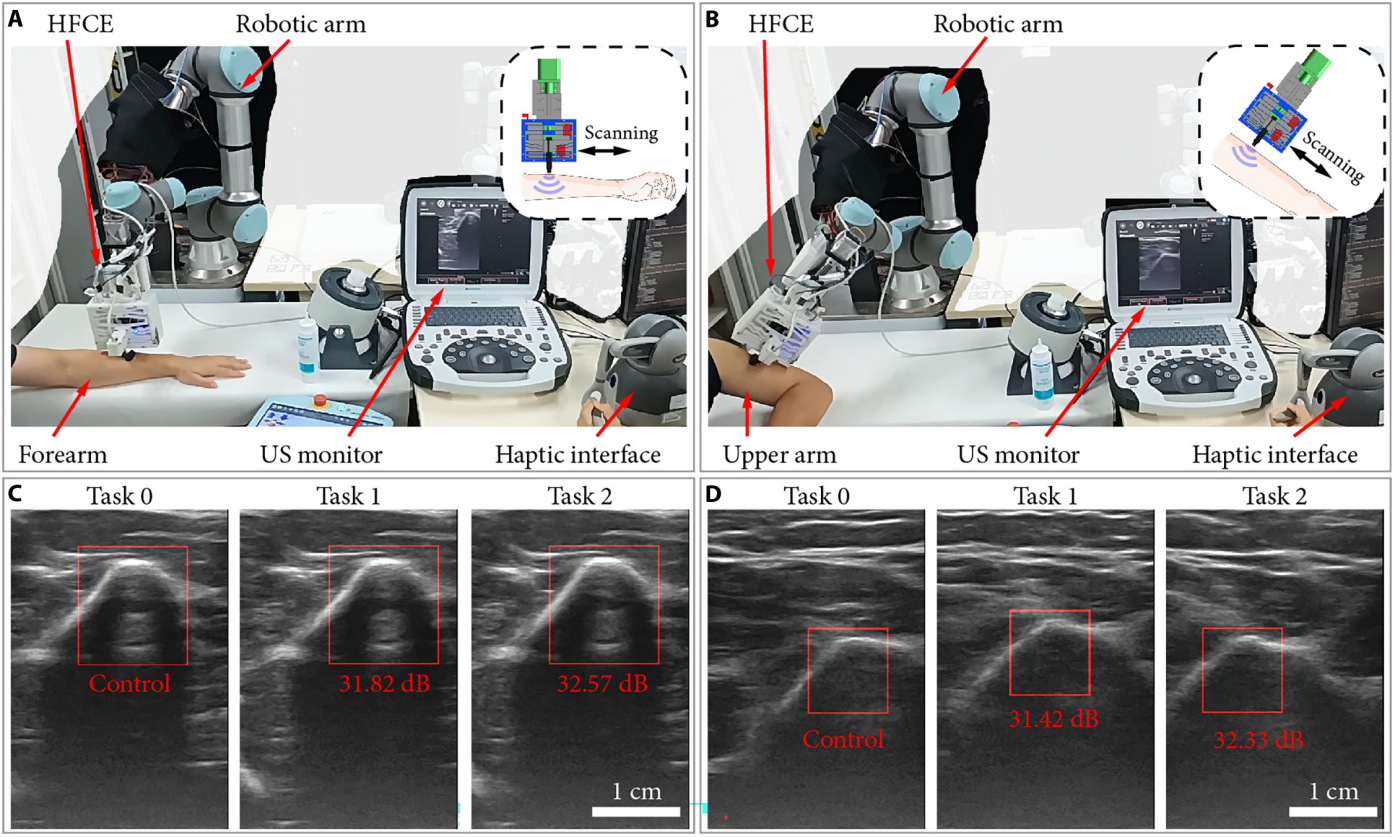
Demonstration of the robotic US scanning system. (A) Experimental snapshots of forearm scanning. (B) Experimental snapshots of upper arm scanning. (C) US images of the forearm scanning by 3 times. (D) US images of the upper arm scanning 3 times. Red text: PSNR values relative to the control image.

To demonstrate the repeatability of collecting US images with the same quality at different times, an experimental study was carried out to scan the forearm and upper arm of different candidates 3 times (Movie S4). The US probe would leave the scanned surface between every 2 scanning times, indicating that the scanning paths of every 2 scanning tasks differ. The experience-free candidate operator controlled the scanning motion of HFCE. In addition, the set force magnitude is 12.33 N ($V_{F}=-$0.7 V, the minimum set force magnitude). Fig. 7A and B shows the snapshots of scanning the forearm and upper arm, respectively.

For each scanning object, 3 US images are selected during the scanning based on their similar features, as shown in Fig. 7C and D. The US image of the first scanning (task 0) is determined as the control image, and its feature is set as the template image. Then, the template matching is utilized to find the feature images of the other 2 US images. Fig. 7C and D shows the results of template matching for scanning the forearm and upper arm, respectively. Furthermore, the peak signal-to-noise ratio (PSNR) values are derived to evaluate the similarity between the feature images in the compared and control images. For both scanning objects, the PSNR values are derived as around 31 to 33 dB. In addition, a PSNR value of 30 to 40 dB indicates that the distortion of image quality is kept within an acceptable range (i.e., distortion can be perceived but acceptable). The results suggest that the proposed HFCE can effectively improve the repeatability of US scanning imaging.

## Discussion

This article presents a robotic US scanning system based on a hybrid active–passive force control method, which can be helpful in modern medical treatment via US imaging. The force control system is composed of an active force control and a passive force control. Concerning passive force control, a constant-force mechanism is designed based on the stiffness matching approach, which is adopted to buffer force variation through its constant-force region. Regarding the active force control, the real-time feedback of deformation and constant-force magnitude is provided by 2 strain gauges integrated into the constant-force mechanism, which is adopted to adjust the output of 2 positioning stages. The proposed force control system offers the merits of both active and passive force control methods. Specifically, it inherits the superior performance of active force control in maintaining stability during long-range, low-frequency displacement variations while simultaneously preserving the superior performance of passive force control in responding to short-range, high-frequency displacement changes. This synergistic combination enables robust force regulation across a wide dynamic range of operating conditions. Experimental results show that it can eliminate the heavy discrepancy in US images induced by the traditional US scanning made by human operators with different experiences. The reported US scanning system is easy to operate, which reduces the operator’s learning cost. Hence, the doctor can focus on the diagnosis. Moreover, our study demonstrates the effectiveness of the hybrid active–passive force control approach, providing an excellent reference for other tasks requiring force control, such as part polishing and physical rehabilitation.

Simulation study and optimization were carried out based on finite element analysis to determine optimal structural parameters of the passive constant-force mechanism. Experimental tests further calibrated the passive constant-force mechanism and strain gauges. The performance and effectiveness of the proposed HFCE were demonstrated by the contact force measurement and US imaging under vibration shaking. To illustrate the operation’s ease and repeatability, an experience-free operator was invited to scan a sample on the shaker and arms of volunteers. Experimental results indicate that the proposed robotic US scanning system enables the operator to operate in a friendly manner and provides a promising solution to collect repeatable and qualified US images.

The required contact force for US imaging depends on the target tissues and the condition of patients (i.e., body mass index). Referring to the literature [39,40], the required force range can be roughly determined as 1.2 to 20 N. It was found that the proposed HFCE cannot cover the needed range of force. However, it is easy to decrease the adjusting constant-force magnitude of the passive constant-force mechanism without preloading displacement by reducing its out-of-plane thickness (Fig. 2B and Fig. S4). Therefore, to cover the required force range, we need 6 passive constant-force mechanisms with 6 scales of out-of-plane thickness, including 0.8 mm (0.8 to 1.4 N), 1.4 mm (1.4 to 2.4 N), 2.3 mm (2.4 to 4.1 N), 4 mm (4.1 to 7.1 N), 6.9 mm (7.1 to 12.3 N), and 12 mm (12.3 to 21.3 N). An additional mechanism is also needed to enlarge the displacement between the US probe and the main structure of the HFCE. This will decrease the performance of force variation buffering to reduce the size of the executive side, which may enhance patient compliance. The path generation of US scanning has also gained great attention. For semiautomatic planning, the predefined path or interested area from the doctor was automatically tuned based on real-time 3D information [41–44]. There are also several fully automatic plannings for different scanned tissues [45,46]. However, the safety and generated duty of fully automatic planning remains controversial. From this perspective, the semiautomatic US scanning system would rapidly assist in medical treatment. In the future, we will integrate the function of semiautomatic planning into our proposed system to further release the burden of doctors. It is also desired to fabricate the HFCE with other ranges for further demonstration of scanning various tissues or organs under the guidance or operation of professional surgeons.

## Data Availability

The data that support the findings of this study are available from the corresponding author upon reasonable request.
