# Supplementary material for: Robotic Ultrasound Scanning End-Effector with Adjustable Constant Contact Force
Source: Cyborg Bionic Syst. 2025 May 2;6:0251. doi: 10.34133/cbsystems.0251 (PMC12046132; doi:10.34133/cbsystems.0251)
Supplement: Supplementary 1 — Supplementary Text Figs. S1 to S4 Table S1 Movies S1 to S4 [file cbsystems.0251.f1.zip › Sup-Info-r1.pdf]

Cyborg and Bionic Systems
A SCIENCE PARTNER JOURNAL
SPJ

Supplementary Materials

Robotic Ultrasound Scanning End-effector with Adjustable Constant Contact Force

*Zehao Wu, Xianli Wang, Yuning Cao, Weijian Zhang, and Qingsong Xu**

Department of Electromechanical Engineering, Faculty of Science and Technology, University of Macau, Taipa, Macau, China
*Address correspondence to: qsxu@um.edu.mo

The PDF file includes:

Supplementary Text: Basic principle of the passive constant-force mechanism

Figure S1. Schematic of the passive constant-force mechanism.

Figure S2. Voltage-deformation relations of the strain gauges.

Figure S3. Complete force-deformation curves under various preloading displacements.

Figure S4. Simulation results of the passive constant-force mechanism with various out-of-plane thickness.

Table S1. Optimal parameters of the passive constant-force mechanism.

Other Supplementary Material for this manuscript includes the following:

Movie S1 (.mp4 format). US images under various contact forces.

Movie S2 (.mp4 format). US images at various noise magnitudes.

Movie S3 (.mp4 format). Experimental study for scanning the sample by robotic US scanning system.

Movie S4 (.mp4 format). US images of the arm scanning by three times.

Supplementary Text

Basic principle of the passive constant-force mechanism

The passive constant-force mechanism in this study is based on the stiffness combination configuration. This approach has the advantages of easy adjustment of structural parameters and easy implementation of changing the constant-force magnitude.

The negative-stiffness mechanism is achieved by four parallel-connected tilted curve beams. According to the buckling effect, the reaction force from the negative-stiffness mechanism (F_n) is determined by the tangential force (F_{nt}), the radial force (F_{nr}) and the tilted angle of the tilted beam (γ), which can be expressed as:

$$F_n = 4F_{nr}(d)cos\gamma + 4F_{nt}(d)sin\gamma \qquad \text{Equation (1)}$$

Then, the mechanical property of the negative-stiffness mechanism can be determined based on its structural parameters and material.

$$F_n\ (E, l_n, w_n, r_n, a_n, t, d) \qquad \text{Equation (2)}$$

where E denotes the Young's modulus of the material. The structural parameters of the negative-stiffness mechanism (l_n, w_n, r_n, a_n, t, d) are shown in figure S1A.

The positive-stiffness mechanism is composed of multiple straight beams. Then, with a preloading displacement (d_F), the reaction force from the positive-stiffness mechanism (F_p) can be derived as:

$$F_p = \frac{Etw_p^3}{l_p^3}(\frac{3}{2}d - d_F) \qquad \text{Equation (3)}$$

where the structural parameters of the negative-stiffness mechanism (l_p, w_p) are shown in figure S1A.

Then, the contact force between the US probe and the scanned tissue (F_c) can be determined as:

$$F_c = F_n + F_p \qquad \text{Equation (4)}$$

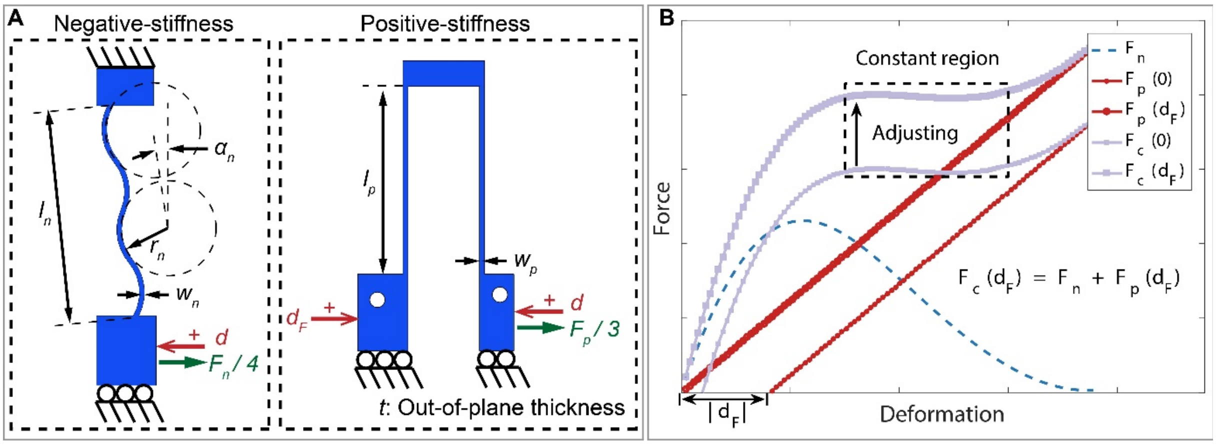

Fig. S1. Schematic of the passive constant-force mechanism. (A) Structural parameters of the passive constant-force mechanism. (B) Force-displacement relations of the constant-force mechanism and its sub-mechanisms.

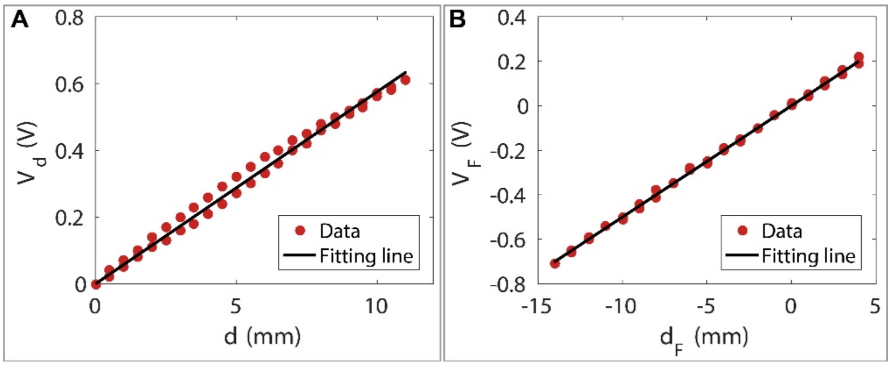

Fig. S2. Voltage-deformation relations of the strain gauges. (A) Voltages at the position (V_d) with various deformations (d). (B) Voltages at the constant force (V_F) with various preloading displacements (d_F).

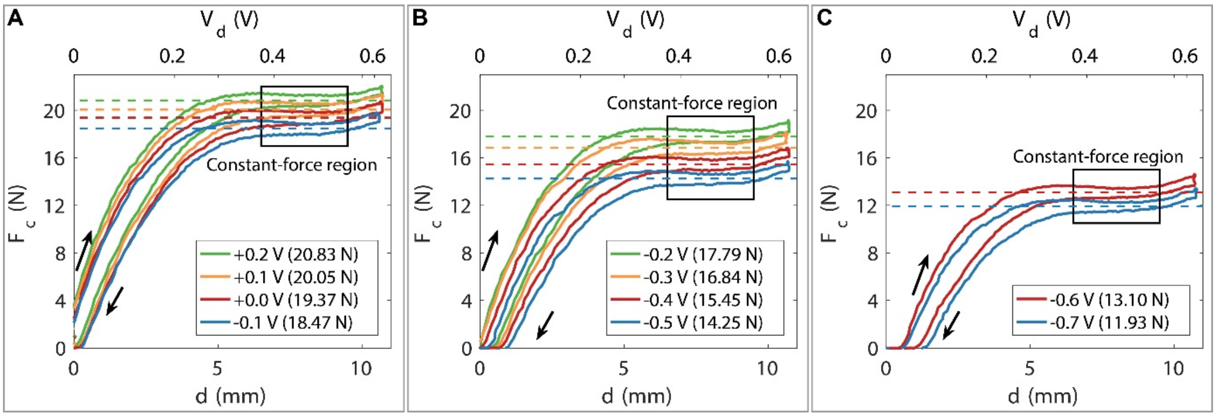

Fig. S3. Complete force-deformation curves under various preloading displacements. (A) Force-deformation curves with V_d of -0.1 to $+0.2$ V. (B) Force-deformation curves with V_d of -0.5 to -0.2 V. (C) Force-deformation curves with V_d of -0.7 to -0.6 V.

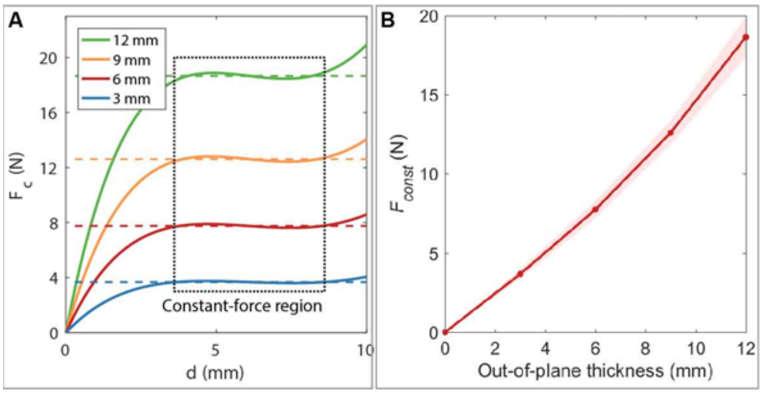

Fig. S4. Simulation results of the passive constant-force mechanism with various out-of-plane thickness and without preloading displacement. (A) Complete force-deformation curves with various out-of-plane thickness. (B) Relationship between the magnitude of constant force and the out-of-plane thickness. Shaded area: force variation in the constant-force region.

Table S1. Optimal parameters of the passive constant-force mechanism.

Parameter	Value	Parameter	Value
$\boldsymbol{l_n}$	45.9 mm	w_n	1.1 mm
$\boldsymbol{r_n}$	9.95 mm	a_n	8.5°
$\boldsymbol{l_p}$	36.7 mm	w_p	1.0 mm
$\boldsymbol{t}$	12.0 mm		
